# Amiodarone as an autophagy promoter reduces liver injury and enhances liver regeneration and survival in mice after partial hepatectomy

**DOI:** 10.1038/srep15807

**Published:** 2015-10-30

**Authors:** Chih-Wen Lin, Yaw-Sen Chen, Chih-Che Lin, Yun-Ju Chen, Gin-Ho Lo, Po-Huang Lee, Po-Lin Kuo, Chia-Yen Dai, Jee-Fu Huang, Wang-Long Chung, Ming-Lung Yu

**Affiliations:** 1 Graduate Institute of Medicine, College of Medicine, Kaohsiung Medical University, Kaohsiung; 2 Division of Gastroenterology and Hepatology, Department of Medicine, E-Da Hospital, I-Shou University, Kaohsiung; 3 Health Examination Center, E-Da Hospital, I-Shou University, Kaohsiung; 4 School of Medicine, College of Medicine, I-Shou University, Kaohsiung; 5 Department of Surgery, E-Da Hospital, I-Shou University, Kaohsiung; 6 Department of Medical Research, E-Da Hospital, I-Shou University, Kaohsiung; 7 Department of Biological Science & Technology, I-Shou University, Kaohsiung; 8 Department of Surgery, Kaohsiung Chang Gung Memorial Hospital and Chang Gung University College of Medicine, Kaohsiung; 9 Hepatobiliary Division, Department of Internal Medicine, Kaohsiung Medical University Hospital, Kaohsiung Medical University, Kaohsiung; 10 Faculty of Medicine, College of Medicine, and Center for Infectious Disease and Cancer Research, and Center for Lipid and Glycomedicine Research, Kaohsiung Medical University, Kaohsiung; 11 Institute of Biomedical Sciences, National Sun Yat-Sen University, Kaohsiung, Taiwan

## Abstract

The deregulation of autophagy is involved in liver regeneration. Here, we investigated the role of autophagy in the regulation of liver regeneration after partial hepatectomy (PHx) and the development of pharmacological interventions for improved liver regeneration after PHx. We show that autophagy was activated in the early stages of liver regeneration following 70% PHx *in vivo*. Moreover, amiodarone was associated with a significant enhancement of autophagy, liver growth, and hepatocyte proliferation, along with reduced liver injury and the termination of liver regeneration due to decreased transforming growth factor-β1 expression after 70% PHx. The promotion of autophagy appeared to selectively increase the removal of damaged mitochondria. We also found that Atg7 knockdown or pretreatment with chloroquine aggravated the liver injury associated with 70% PHx and reduced liver growth and hepatocyte proliferation. Finally, amiodarone improved liver regeneration, survival, and liver injury after 90% PHx. In conclusion, our results indicate that autophagy plays an important role in mouse liver regeneration and that modulating autophagy with amiodarone may be an effective method of improving liver regeneration, increasing survival, and ameliorating liver injury following PHx.

The liver is an organ that has a remarkable capacity for self-regeneration^1^,^2^. Liver regeneration in rodents that have had 70% of their liver removed in a partial hepatectomy (PHx) has become a useful model for studying regenerative organ growth. Hepatocytes that are typically in a quiescent phase enter the cell cycle following PHx. These hepatocytes then proliferate, restoring both normal hepatic mass and functional capacity^1^,^2^,^3^,^4^. Stimulating liver regeneration is a potential strategy for the treatment of chronic liver diseases, PHx, split-liver transplantation, and living-donor liver transplantation^5^. The enhancement of liver regeneration is also required for the success of pharmacological approaches that aim to reverse hepatic fibrosis due to chronic liver diseases^6^. Although liver regeneration offers curative options, postoperative complications are common if the remnant liver or graft is either too small or too low-quality to maintain adequate organ function^5^,^6^,^7^. Thus, effective liver regeneration is a major goal in the clinic.

Macroautophagy (referred to here as autophagy) is a process through which long-lived proteins and damaged organelles are conveyed to the lysosome for removal by degradation and recycling^8^,^9^,^10^. Research has shown that autophagy plays a significant role in the pathogenesis of numerous human diseases and is induced by various stress conditions. Autophagy plays an active role in liver physiology and pathogenesis^8^,^9^,^10^. In liver diseases, autophagy can also serve as an effective defense mechanism against numerous pathological stresses, such as alcohol- and non-alcohol-induced fatty-liver conditions, hepatic alpha1-antitrypsin mutant proteins, and liver fibrosis^11^,^12^,^13^,^14^,^15^,^16^,^17^. Liver-specific autophagy-related gene 7 (Atg7)-knockout mice exhibit accumulation of peroxisomes, deformed mitochondria, and abnormal membrane structures, resulting in hepatomegaly and liver injury^18^. Liver-specific Atg5-knockout mice exhibit reduced hepatocyte senescence and energy requirements during liver regeneration^19^.

Numerous agents can stimulate autophagy *in vitro*; however, to date, few have been tested *in vivo*^20^,^21^,^22^,^23^. Amiodarone is a widely used, well-tolerated anti-arrhythmic drug in the clinic. It can decrease calcium permeability, which can induce autophagy^21^,^22^,^23^. Chloroquine (CQ), an anti-malarial lysosomotropic agent and a potential anti-cancer agent, has been identified as an autophagy inhibitor at a later step in the pathway, leading to a disruption of lysosome-autophagosome fusion and lysosomal protein degradation^20^,^21^,^22^,^23^,^24^,^25^.

To date, the significance of autophagy in liver regeneration following PHx surgery remains unclear. Accordingly, we conducted this study to explore the role of autophagy in liver regeneration after PHx in mice and to evaluate the pharmacological effects of amiodarone on autophagy-related liver regeneration and survival following PHx. We also inhibited autophagy through Atg7 knockdown and pretreatment with CQ to investigate their suppressive effect on liver growth and hepatocyte proliferation and its aggravating effect on liver injury following PHx. We have explored the important protective role of autophagy in the regeneration of the mouse liver following PHx. Our findings indicate that amiodarone is a promising intervention to promote autophagy, liver regeneration, hepatocyte proliferation and survival, and reduced liver damage following PHx.

## Results

### Activation of autophagy in the early phase of liver regeneration following PHx

**Figure 1:**
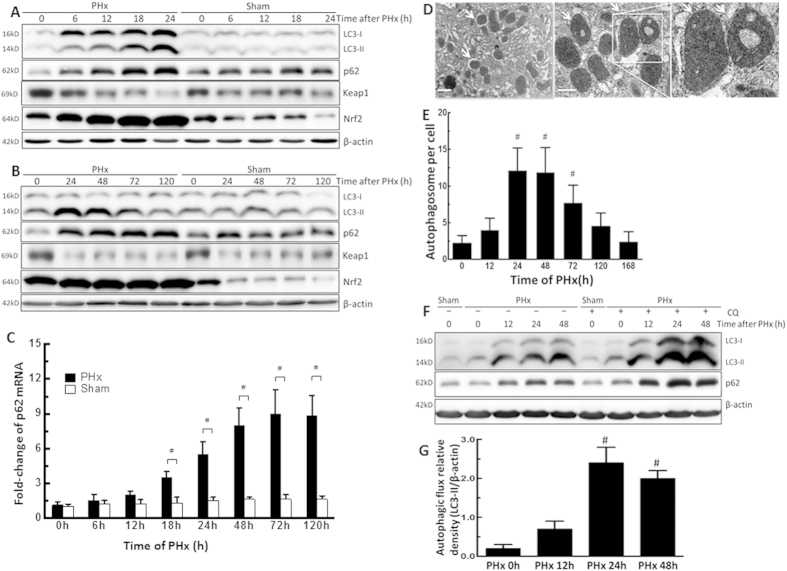
PHx induced autophagy and increased autophagic flux in the early phase of liver regeneration. Wild-type mice were treated with PHx or a sham operation and then sacrificed at 0–168 h after surgery. (**A,B**) Liver tissues were harvested, and tissue extracts were analyzed for LC3-II, p62, Keap1, Nrf2, and β-actin protein levels at each time point by Western blotting. (**C**) Fold-change in p62 mRNA expression. (**D**) Electron microscopic images of autophagosomes in the liver regeneration. Arrows indicate autophagosomes with double-membrane structures containing with mitochondria or cytosolic contents. Scale bar, 1 μm. (**E**) The number of autophagosomes in hepatocytes at the different time points. (**F,G**) Wild-type mice were intraperitoneally injected with or without chloroquine (CQ) at 0.5 h before either the sham operation or PHx and then once per day until 48 h. Liver tissues were harvested at 0 h, 12 h, 24 h, and 48 h after surgery, and tissue extracts were analyzed for LC3-II, p62, β-actin and autophagic flux. Autophagic flux was quantified by subtracting LC3-II levels in mice without additional CQ treatment from the LC3-II levels in mice with additional CQ treatment. The 0 h-PHx condition without CQ treatment was set to represent an autophagic flux of 100%. The values are shown as the mean ± SD in the bar graph and compared by Student’s t test. #*P* < 0.05 versus 0 h-treated PHx (n = 6).

To determine the predominant factor of autophagy in liver regeneration following 70% PHx, we first examined the effect of autophagy on the dynamic changes that occur in the early phase of liver regeneration. The autophagy-related proteins LC3-II and p62 were measured after PHx. Lipidated LC3 (LC3-II) is an autophagosome marker^26^,^27^,^28^. p62 (SQSTM1) is degraded by autophagosome-lysosomal fusion. LC3-II protein levels showed a substantial increase at 6 to 72 h after PHx compared with their counterpart control groups (Fig. 1A,B). LC3-II protein levels also increased at 6 h, peaking from 24 h to 72 h, and returning to baseline at 120 h after PHx (Fig. 1A,B). Protein levels of p62, which regulates ubiquitin-positive aggregates during autophagic deficiency, were slightly higher compared with their counterpart control groups throughout the entire study period (Fig. 1A,B). The LC3-II and p62 protein levels did not change with time in the sham-operated mice (Fig. 1A,B). Furthermore, Kelch-like ECH-associated protein 1 (Keap1) levels were slightly decreased, and nuclear factor erythroid 2-related factor 2 (Nrf2) levels were significantly increased after PHx (Fig. 1A,B). Nrf2 levels were significantly higher in PHx groups compared with their counterpart control groups (Fig. 1A,B). The p62 mRNA levels gradually increased and were significantly higher in PHx groups compared with their counterpart control groups (Fig. 1C). These results indicated that the increase in p62 mRNA levels might have been modulated by compensatory Nrf2 activation after PHx. Moreover, the number of autophagosomes observed by electron microscopy (EM) was significantly increased after PHx, peaking from 24 h to 72 h, and returning to baseline at 168 h (Fig. 1D,E). In addition, pretreatment with CQ in PHx-treated mice blocked the degradation of autophagosomes, with further elevated LC3-II protein levels at 24 h and 48 h, indicating that PHx persistently increased autophagic flux (Fig. 1F,G). p62 was degraded during PHx and induced by CQ (Fig. 1F). Taken together, these observations indicated that PHx induced autophagy in the early phase of liver regeneration.

### Enhanced autophagy and increased autophagic flux by amiodarone during liver regeneration after PHx

**Figure 2:**
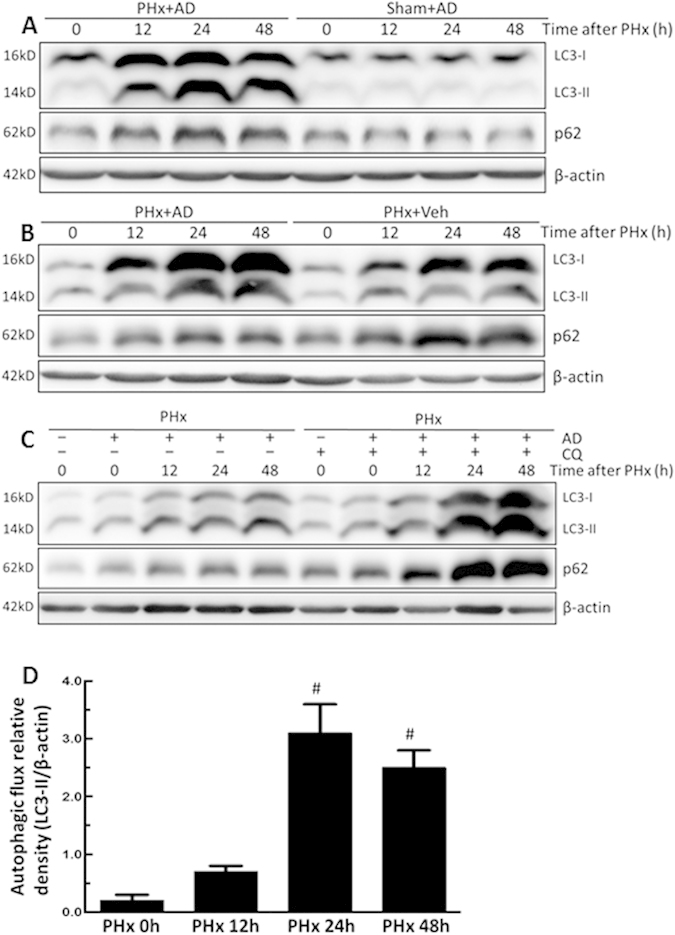
Amiodarone induced autophagy and increased autophagic flux in the early phase of liver regeneration after PHx. Wild-type mice were intraperitoneally injected with vehicle (Veh), amiodarone (AD), or chloroquine (CQ) at 0.5 h before PHx or the sham operation and then once per day until 48 h. Liver tissues were harvested at 0 h, 12 h, 24 h, and 48 h after surgery, and the tissue extracts were analyzed for LC3-II, p62, and β-actin protein by Western blotting (**A,B**). Autophagic flux was quantified by subtracting LC3-II levels in mice without additional CQ treatment from the LC3-II levels in mice with additional CQ treatment. The 0 h-PHx condition without CQ treatment was set to represent an autophagic flux of 100% (**C,D**). Relative expression protein levels of LC3-II were calculated as the optical densities of their blots normalized to the β-actin blots, and the densitometric values are shown. The values are shown as the mean ± SD in the bar graph and compared by Student’s t test. #*P* < 0.05 versus 0 h-treated PHx (n = 6).

To investigate the effect of pharmacological modulation of autophagy on liver regeneration, mice were treated with amiodarone 30 min before PHx. LC3-II protein levels were significantly increased at 12 h, 24 h, and 48 h after PHx among mice pretreated with amiodarone compared with the sham-operated mice (Fig. 2A). This difference was not observed among the sham-operated mice pretreated with amiodarone (Fig. 2A). Furthermore, LC3-II protein levels also increased at 12 h, 24 h, and 48 h after PHx among mice pretreated with amiodarone compared with the vehicle-treated PHx mice (Fig. 2B). These findings indicated that amiodarone might induce a significant increase in autophagosomes by either increasing autophagosome formation or accumulation. Pretreatment with CQ in amiodarone-treated PHx mice revealed a blocked degradation of autophagosomes with further elevated LC3-II protein levels at 24 h and 48 h, indicating that amiodarone-treated PHx mice persistently showed significantly increased autophagic flux compared with 0 h-treated PHx mice (Fig. 2C,D). These results indicated that amiodarone induced autophagy through increased autophagosome formation and degradation. Furthermore, p62 expression was detected, further confirming that amiodarone induced a complete induction of autophagy after PHx through autophagosome formation and its degradation by lysosomes. p62 protein levels were slightly increased in amiodarone-treated PHx mice compared with sham-operated mice (Fig. 2A). p62 protein levels were decreased in amiodarone-treated PHx mice compared with vehicle-treated PHx mice (Fig. 2B). p62 protein levels were significantly decreased in amiodarone-treated PHx mice without CQ compared with amiodarone-treated PHx mice with CQ (Fig. 2C). These observations further supported the conclusion that amiodarone induced autophagy in the early phase of liver regeneration following PHx surgery.

### Enhanced autophagy after PHx by amiodarone promoted liver growth and hepatocyte proliferation

**Figure 3:**
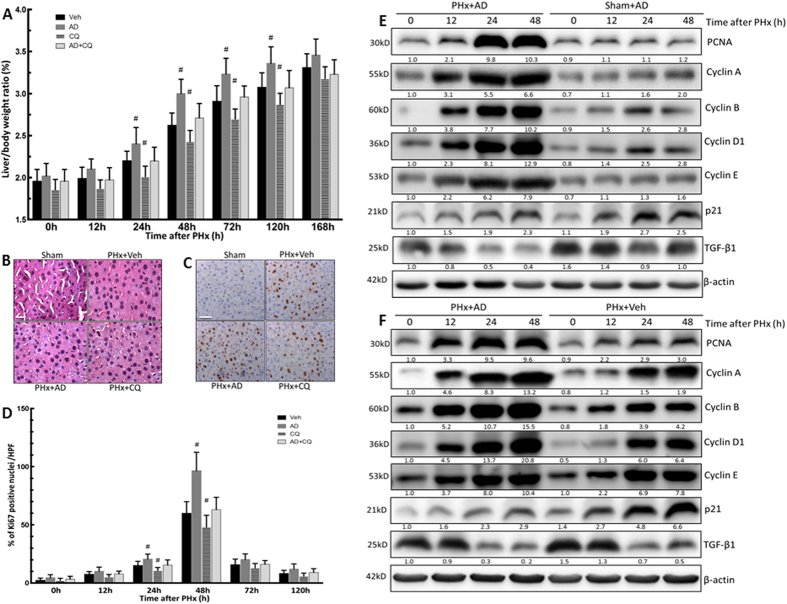
Amiodarone enhanced liver growth and hepatocyte proliferation in the liver regeneration after PHx. Wild-type mice were intraperitoneally injected with vehicle (Veh), amiodarone (AD), or chloroquine (CQ) at 0.5 h before PHx or sham operation and then once per day until 168 h. Liver tissues were harvested at 0–168 h after surgery. Liver-to-body-weight ratios were calculated (**A**). Liver sections at 24 h after the sham operation, PHx with Veh, AD, or CQ were stained with H&E; original magnification, 400X (**B**). Representative immunohistochemical staining of Ki67 is shown (**C**). The percentage of Ki67-positive nuclei in hepatocyte was counted under low-power fields (200 ×) in 15 random sections from at least six different mice (**D**). Liver tissues were harvested at 0 h, 12 h, 24 h, or 48 h after surgery, and the tissue extracts were analyzed for PCNA, cyclin A, B, D1, E, p21, TGF-β1, and β-actin protein by Western blotting (**E,F**). The values are shown as the mean ± SD in the bar graph and compared by Student’s t test. #*P* < 0.05 versus vehicle-treated PHx. Scale bar, 25 μm in B, 50 μm in C (n = 6).

To determine whether autophagy played a functional role in the control of hepatocyte proliferation in the liver following PHx, an event crucial for liver regeneration, mice were treated with amiodarone before PHx. The liver-to-body-weight ratio was significantly increased in amiodarone-treated PHx mice compared with the counterpart vehicle-treated PHx mice at 24 h, 48 h, 72 h, and 120 h (Fig. 3A). There was neither inflammatory liver cell infiltration nor necrosis in amiodarone-treated groups, as determined using hematoxylin and eosin (H&E) staining (Fig. 3B). The percentage of Ki67-positive hepatocyte nuclei determined through immunohistochemical (IHC) staining revealed significant hepatocyte proliferation in PHx mice in the vehicle and amiodarone groups compared with sham-operated mice. A significant increase in Ki67-positive nuclei was found in amiodarone-treated PHx mice compared with their counterpart vehicle-treated PHx mice at 24 h and 48 h after PHx, with a peak at 48 h (Fig. 3C,D). Hepatic protein expression levels of PCNA (a marker of hepatocyte proliferation) and cyclins A, B, D1, and E (which are involved in the regulation of cyclin-dependent kinases) were significantly higher in amiodarone-treated PHx mice than the sham-operated or vehicle-treated PHx mice at 12 h, 24 h, and 48 h (Fig. 3E,F). The protein levels of p21, a potent cyclin-dependent kinase inhibitor, were significantly lower in amiodarone-treated PHx mice than in the sham-operated or vehicle-treated PHx mice (Fig. 3E,F). In addition, TGF-β1 is known to be involved in liver regeneration termination through its antiproliferative activity^2^. We observed that amiodarone-treated PHx mice had significantly lower TGF-β1 protein levels than the sham-operated or vehicle-treated PHx mice (Fig. 3E,F). The liver-to-body-weight ratio, Ki67, PCNA, cyclin A, B, D1, and E, p21, and TGF-β1 protein levels did not differ significantly from sham-operated mice with different treatments. Taken together, we demonstrated that the enhancement of autophagy by amiodarone led to an increase in hepatocyte proliferation and liver growth and a decrease in the termination of liver regeneration after PHx.

### Inhibition of autophagy reduced liver growth and hepatocyte proliferation in the early phase of liver regeneration following PHx

**Figure 4:**
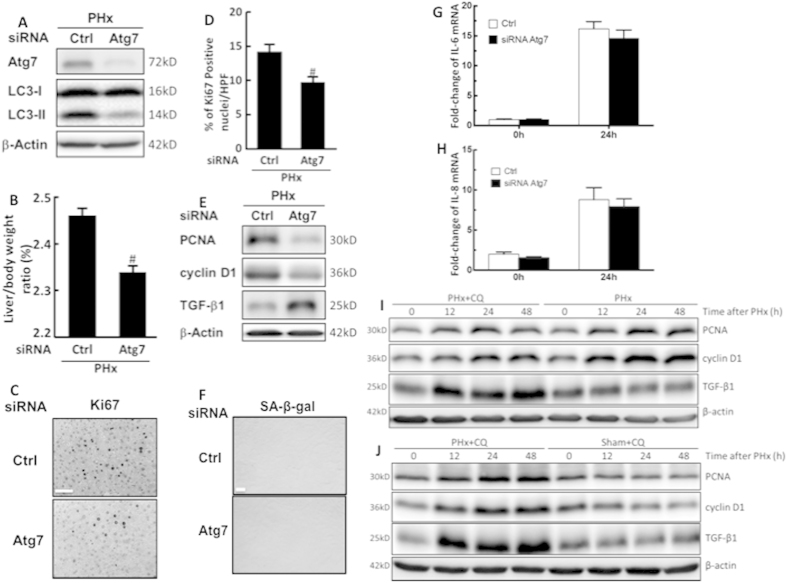
Inhibition of autophagy reduced liver growth and hepatocyte proliferation in the early phase of liver regeneration after PHx. Wild-type mice were given control or Atg7-specific siRNA for 48 h before treatment with PHx. Liver tissues were harvested at 24 h after surgery and tissue extracts were analyzed for Atg7, LC3-II, and β-actin by Western blotting (**A**). The liver-to-body-weight ratios were calculated (**B**). Representative immunohistochemical staining of Ki67 is shown. Scale bar, 50 μm (**C**). The percentage of Ki67-positive nuclei in hepatocytes was counted in low-power field (200X) in 15 random sections from 3 different mice (**D**). Tissue extracts were analyzed for PCNA, cyclin D1, TGF-β1, and β-actin by Western blotting (**E**). Immunohistochemical staining of senescence-associated β-galactosidase (SA-β-gal) in hepatocytes. Scale bar, 100 μm (**F**). Fold-changes in IL-6 (**G**) and IL-8 (**H**) mRNA expression at 24 h after 70% PHx. Wild-type mice were intraperitoneally injected with vehicle (Veh) or chloroquine (CQ) at 0.5 h before PHx or the sham operation and then once per day until 48 h. Liver tissues were harvested at 0–48 h after surgery and tissue extracts were analyzed for PCNA, cyclin D1, TGF-β1, and β-actin by Western blotting (**I,J**). The values are shown as the mean ± SD in the bar graph and compared using Student’s t test. #P < 0.05 versus control-treated PHx (n = 3).

To inhibit autophagy, mice were pretreated with a specific siRNA against Atg7, which effectively knocked down Atg7 expression in the liver with a subsequent decrease of LC3-II level in PHx mice (Fig. 4A). The inhibition of autophagy by knocking down Atg7 resulted in a significant decrease in liver-to-body-weight ratio, hepatocyte proliferation, and protein levels of PCNA and cyclin D1, along with a significant increase in TGF-β1 protein levels in PHx mice (Fig. 4B–E). However, the levels of senescence-associated β-galactosidase (SA-β-gal), interleukin (IL)-8, and IL-6 in hepatocytes at 24 h after PHx showed no significant differences in PHx mice between the Atg7-knockdown and control groups (Fig. 4F–H). Moreover, a significantly lower liver-to-body weight ratio (Fig. 3A), fewer Ki67-positive hepatocyte nuclei (by IHC staining) (Fig. 3C,D), lower protein levels of PCNA and cyclin D1, and higher TGF-β1 protein levels (Fig. 4I) were observed in CQ-treated PHx mice compared with the vehicle-treated PHx mice. Moreover, an increase in the protein levels of PCNA, cyclin D1, and TGF-β1 were observed in CQ-treated PHx mice compared with the sham-operated mice (Fig. 4J). Taken together, we demonstrated that inhibition of autophagy by Atg7 knockdown or pretreatment with CQ resulted in a significant decrease in liver growth because hepatocyte proliferation and cell cycle progression were suppressed, and the termination of early-phase liver regeneration increased following PHx.

### Enhanced autophagy alleviated liver injury after PHx

**Figure 5:**
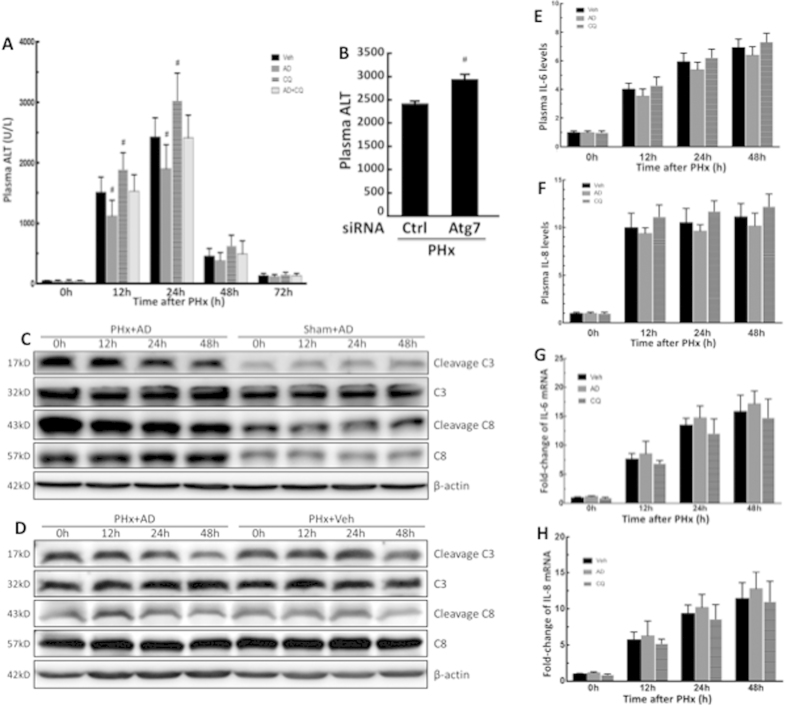
Amiodarone alleviated liver injury after PHx. Wild-type mice were intraperitoneally injected with vehicle (Veh), amiodarone (AD), or chloroquine (CQ) at 0.5 h before PHx or sham operation and then once per day until 72 h. Plasma samples were collected at 0–72 h after PHx and analyzed for ALT levels (**A**). Wild-type mice were given control or Atg7-specific siRNA before PHx and plasma samples were analyzed for ALT levels at 24 h (**B**). Liver tissues were harvested at 0–48 h after surgery, and Caspase-8 cleavage and Caspase-3 cleavage was assessed by Western blot analysis (**C**,**D**). Samples were collected at 0–48 h after PHx and analyzed for IL-6 and IL-8 levels in plasma (**E,F**) and hepatocytes (**G**,**H**). The values are shown as the mean ± SD in the bar graph and compared by Student’s t test; #*P* < 0.05 versus vehicle-treated PHx (n = 6).

Partial hepatectomy-induced liver injury caused an increase in plasma ALT levels, whereas mice pretreated with amiodarone before PHx showed significantly decreased plasma ALT levels at 12 h and 24 h compared with the vehicle-treated PHx mice. By contrast, pretreatment of mice with CQ before PHx significantly increased plasma ALT levels compared with vehicle-PHx mice (Fig. 5A). Similarly, knockdown of Atg7 in mice by siRNA revealed an increase in plasma ALT levels after PHx (Fig. 5B). Our findings confirmed that autophagy does play an important role in reducing liver injury in the early phase of liver regeneration following PHx. However, both Caspase-3 cleavage and Caspase-8 cleavage were slightly lower in amiodarone-treated PHx mice compared with 0 h-treated PHx mice (Fig. 5C). The expression levels of Caspase-3, cleaved Caspase-3, Caspase-8, and cleaved Caspase-8 were significantly higher in amiodarone-treated PHx mice compared with sham-operated mice (Fig. 5C). The levels of cleaved Caspase-3 and Caspase-8 were not significantly different in amiodarone-treated PHx mice compared with vehicle-treated PHx mice (Fig. 5D). IL-6 and IL-8 levels in plasma and hepatocytes were lower but not significantly different in amiodarone-treated PHx mice compared with vehicle-treated PHx mice at different time points (Fig. 5E–H). Moreover, IL-6 and IL-8 levels in plasma and hepatocytes were significantly higher in 12, 24, and 48 h-treated PHx mice compared with 0 h-treated PHx mice (Fig. 5E–H).

### Amiodarone induced autophagy via mTOR-independent signaling after PHx

**Figure 6:**
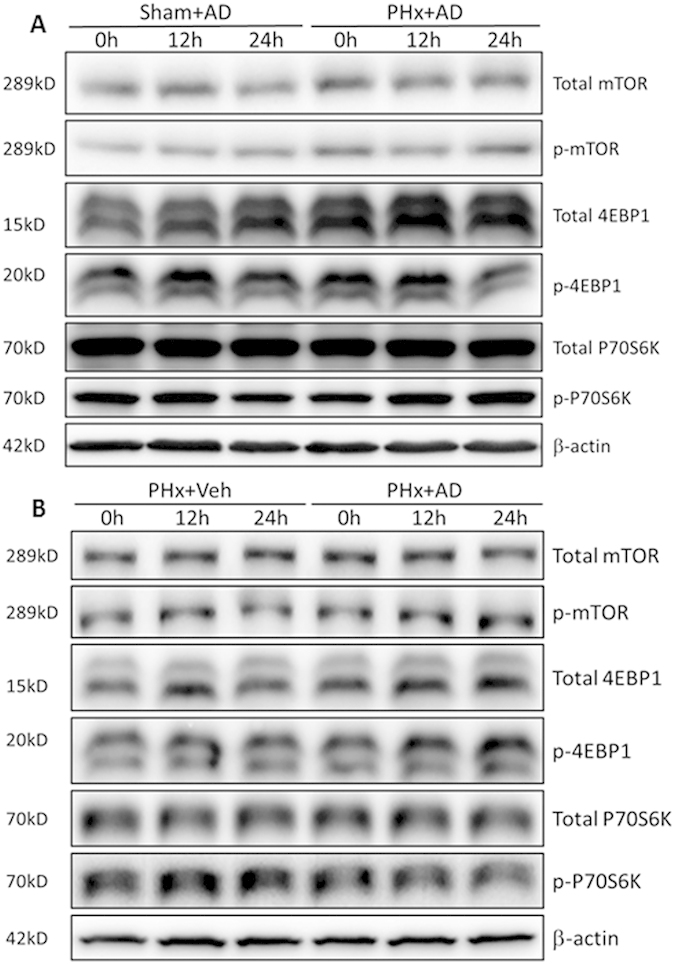
Amiodarone induced autophagy via mTOR-independent signaling after PHx. Wild-type mice were intraperitoneally injected with vehicle (Veh) or amiodarone (AD) at 0.5 h before PHx or the sham operation. Liver tissues were harvested at 0 h, 12 h, and 24 h after surgery and the total and phosphorylation status of mTOR, 4EBP1, and p70 S6K was assessed by Western blot analysis (**A**,**B**) (n = 6).

We further investigated whether amiodarone induced autophagy via mTOR-independent signaling by Western blotting. No difference was found in the protein levels of phosphorylated and total mTOR, phosphorylated and total 4-EBP1, and phosphorylated and total p70 S6 Kinase among amiodarone-treated mice, their counterpart sham-operated mice, and their counterpart vehicle-treated PHx mice at 0 h, 12 h, and 24 h (Fig. 6A,B). These findings indicated that amiodarone induced autophagy via mTOR-independent signaling following PHx.

### Promotion of autophagic activity increased the removal of damaged mitochondria

**Figure 7:**
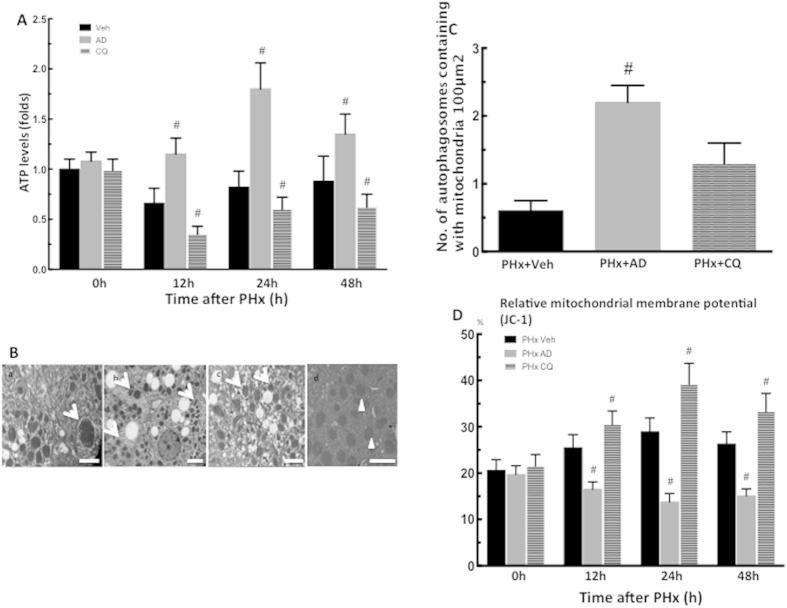
Amiodarone increased removal of damaged mitochondria. Wild-type mice were intraperitoneally injected with vehicle (Veh), amiodarone (AD), or chloroquine (CQ) at 0.5 h before PHx or sham operation and then once per day until 48 h. Liver tissues were harvested at 0–48 h after surgery. The hepatic ATP concentrations were collected at 0–48 h after PHx (**A**). Electron microscopic images of autophagosomes containing with mitochondria in PHx mice treated with Veh (a), AD (b), and CQ (c)and the quantification of autophagosomes containing with mitochondria at 24 h after PHx. Arrows indicate autophagosomes containing with mitochondria and arrow heads indicate no structures representing mitochondria itself (d). Scale bar, 2 μm (**B,C**). Proportion of hepatocytes with a low mitochondrial membrane potential was collected at 0–48 h after PHx (**D**). The values are shown as the mean ± SD in the bar graph and compared by Student’s t test; #*P* < 0.05 versus vehicle-treated PHx (n = 6).

Hepatic ATP levels were significantly higher in amiodarone-treated PHx mice compared with vehicle-treated PHx mice (Fig. 7A). The EM analysis showed that a notable fraction of hepatic autophagosomes contained mitochondria, which was further increased in PHx mice by pretreatment with amiodarone (Fig. 7B). The quantification of hepatocyte mitochondria fragmentation was significantly higher in amiodarone-treated PHx mice compared with vehicle-treated PHx mice (Fig. 7B,C). Furthermore, the proportion of hepatocytes with a low mitochondrial membrane potential (*ΔΨm*) was lower in amiodarone-treated PHx mice compared with vehicle-treated PHx mice (Fig. 7D). Taken together, these findings indicated that activation of autophagy by amiodarone maintains hepatic ATP levels through the immediate removal of damaged mitochondrial by a selective degradation system after PHx.

### Amiodarone improved mouse survival after 90% massive hepatectomy

**Figure 8:**
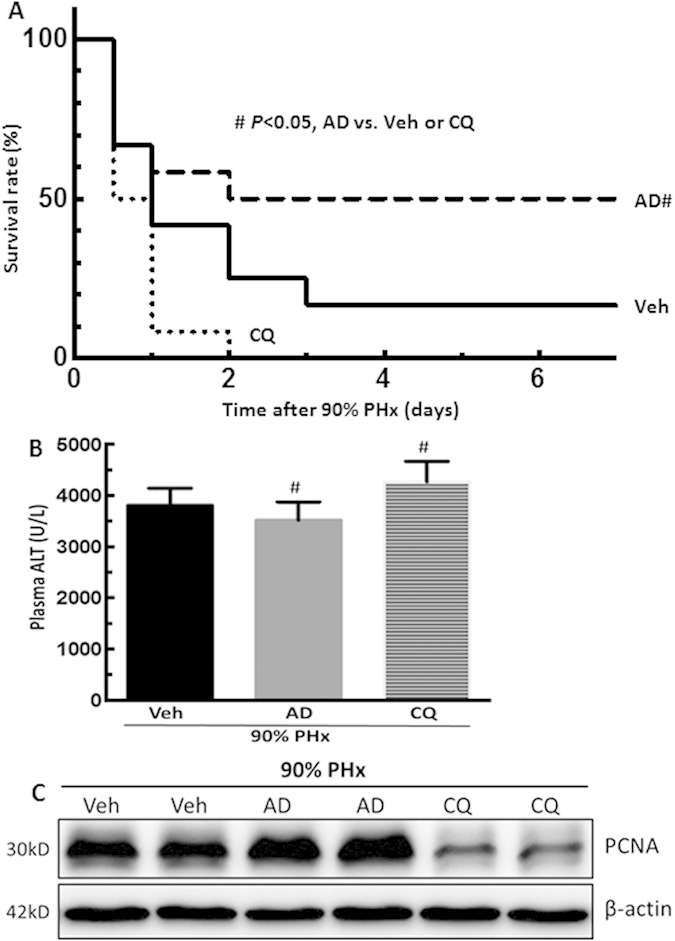
Amiodarone increased the survival rate following 90% massive hepatectomy. Wild-type mice were intraperitoneally injected with vehicle (Veh), amiodarone (AD), or chloroquine (CQ) at 0.5 h before 90% PHx and then once per day until 168 h. (**A**) The survival rate was measured until 7 days after surgery (n = 12). (**B**) Serum ALT levels at 24 h after surgery (n = 6). (**C**) Liver tissues were harvested at 24 h after surgery and expression of PCNA protein was measured by Western blotting analysis. The values are shown as the mean ± SD in the bar graph and compared by Student’s t test. #*P* < 0.05 versus vehicle-treated PHx.

Finally, we investigated the therapeutic effect of amiodarone or CQ on liver regeneration using a 90% massive hepatectomy model. The 7-day survival rate was 50%, 17%, and 0% in amiodarone, vehicle, and CQ-treated mice, respectively, after 90% PHx (Fig. 8A, amiodarone-treated mice versus vehicle-treated or CQ-treated mice, *P* < 0.05). All of the CQ-treated mice died within 2 days after PHx. Amiodarone improved, but CQ aggravated, liver injury after 90% PHx (Fig. 8B). Amiodarone enhanced, but CQ reduced, hepatocyte proliferation in the regenerative liver after 90% PHx (Fig. 8C). Taken together, these findings suggested that enhanced autophagy by amiodarone promoted liver regeneration, reduced liver injury, and improved mouse survival after 90% massive hepatectomy.

## Discussion

Pharmacologic stimulation of liver regeneration is vital for the survival of the PHx recipient and the donor in living donor transplantation^5^,^6^. This study showed that autophagy played an important protective role following PHx *in vivo* in the regulation of liver regeneration and liver injury. We found that autophagy was activated in the early stages of liver regeneration following PHx *in vivo*. Furthermore, the use of pharmacological approaches such as amiodarone led to a marked increase in autophagy, liver growth, hepatocyte proliferation, and survival, and alleviated injury in the early phase of liver regeneration after PHx. By contrast, we showed that in mice, Atg7 knockdown and pretreatment with CQ inhibited autophagy resulted in reduced liver growth and hepatocyte proliferation and increased early-phase termination of regeneration of the post-injury liver after PHx. We conclude that the use of pharmacological agents to modulate autophagy can be a promising approach to improve post-PHx liver recovery. This effective strategy for promoting liver regeneration may decrease hepatic failure and mortality in important clinical conditions such as PHx, split-liver transplantation, and living-donor liver transplantation.

Autophagy involves autophagosome formation, autophagosome-lysosome fusion, and the degradation of unwanted organelles. The process depends on the achievement of all of these phases for the assimilation of dysfunctional organelles or proteins. LC3-II protein, an autophagosome marker, is recruited to form autophagosomes; it remains there until autophagosomal fusion with lysosomes occurs^26^,^27^,^28^. We also know that p62 (confined to the autophagosome formation site) directly interacts with LC3, and then p62 is degraded by autophagosome-lysosome fusion. Thus, impaired autophagosome-lysosome fusion is accompanied by the accumulation of p62^26^. Our study showed a progressive increase in the levels of LC3-II protein in liver regeneration at 6 h, which peaked at 24 h and were maintained until 72 h but returned to baseline at 120 h after PHx. Moreover, p62 showed a slight, gradual increase throughout the study period. However, the increase in p62 might have been due to a compensatory Nrf2 activation after PHx^29^,^30^,^31^. Previous studies have shown that p62 is transcriptionally induced upon oxidative stress by Nrf2 by activation augmentation of liver regeneration and direct binding to an antioxidant response element in the p62 promoter after PHx^29^,^30^,^31^. At the same time, p62 docks directly onto the Keap1, thereby blocking the binding between the Keap1 and Nrf2 that leads to ubiquitylation and degradation of the transcription factor^30^. Indeed, p62 was degraded during PHx and induced by CQ. The autophagic flux also showed a significant increase at 24 h and 48 h after PHx. This observation suggests that following PHx, further increases in LC3-II protein may occur due to an up-regulation of autophagy *per se* in the early phase of liver regeneration.

Amiodarone is a widely used anti-arrhythmic drug that is well tolerated in clinical patients for long-term use, although some patients experience side effects, including pulmonary complications and thyroid disease, under long-term treatment. Amiodarone can decrease calcium permeability and increase potassium permeability, which could also induce autophagy via mTOR-independent signaling^21^,^22^. Moreover, amiodarone is a potential drug to treat HCC through the modulation of autophagy to decrease oncogenic miR-224 expression^23^. Thus, to test the hypothesis that autophagy enables enhanced liver regeneration, in our study, autophagy was induced in mice that had undergone PHx using amiodarone. We found that the autophagic process was induced by increasing LC3-II and autophagic flux and then decreasing p62 levels in PHx mice treated with amiodarone. Furthermore, amiodarone effectively induced autophagy, increased cell cycle progression, increased the removal of damaged mitochondria, led to increased hepatocyte proliferation, promoted regenerative liver growth, and improved survival after PHx. These protective effects were also associated with decreased liver injury and decreased termination of liver regeneration by decreasing TGF-β1 in PHx mice treated with amiodarone. Furthermore, enhanced autophagy by amiodarone promoted liver regeneration, reduced liver injury, and improved mouse survival after 90% massive hepatectomy. These findings provide added support for the hypothesis that autophagy provides an important protective response in liver regeneration after PHx and that activation and completion of the entire process is essential for protection in the regenerative liver. The use of amiodarone could thus induce a beneficial mechanism in hepatocyte proliferation and liver growth during regeneration, possibly via mTOR-independent signaling and through the up-regulation of the complete process of autophagic flux. Indeed, pharmacological enhancement of autophagy by amiodarone could be a novel strategy for promoting liver regeneration, hepatocyte proliferation, and survival. To our knowledge, this finding has not previously been reported in the literature.

Although amiodarone is potential therapeutic drug for increasing liver regeneration, the dosage and time of amiodarone treatment may affect disease development and need to be further investigated. Moreover, there are several well-tolerated antihypertensive drugs, such as verapamil and nimodipine, which have already been shown to stimulate autophagy *in vitro*^21^,^32^. Further research is needed to investigate their potential for enhancing autophagy and their effects in liver regeneration. The use of pharmacological agents to modulate autophagy for liver regeneration has the potential to be an effective therapeutic option that exploits existing medications known to have high safety profiles.

Previous studies have shown that the knockdown of autophagy genes and the complete impairment of autophagosome-lysosome fusion by CQ in the mouse liver are connected to different pathological conditions, including liver disease, alcoholic steatotic liver disease and non-alcoholic steatotic liver disease^13^,^14^,^19^,^28^. Pharmacological inhibition of autophagy by CQ has been used to treat breast cancer and pancreatic adenocarcinoma in clinical trials^25^. Here, we report that inhibition of autophagy by knocking down Atg7 or by pretreatment with CQ resulted in a significant decrease in liver growth because hepatocyte proliferation and cell cycle progression was suppressed, and termination of the early phase of liver regeneration was increased following PHx. Moreover, inhibition of autophagy aggravates liver injury and increases the termination of liver regeneration after PHx. These findings demonstrate that pharmacological inhibition of autophagy by CQ indeed impaired liver regeneration and liver injury after PHx.

In conclusion, we have demonstrated that hepatic autophagy plays a critical role in promoting liver regeneration, diminishing liver injury and prolonging mouse survival after PHx. The pharmacological modulation of autophagy with amiodarone effectively improved liver regeneration, hepatocyte generation, and survival and alleviated liver injury in the mouse liver after PHx.

## Materials and Methods

### Animals

Male C57BL/6 mice were obtained from BioLASCO Taiwan Co., Ltd., and kept at a controlled temperature of 22 ± 1 °C, a relative humidity of 55 ± 5%, and with a 12 h light/12 h dark cycle for 1 week before experiments. All of the animals were housed in the animal facility of the E-Da Hospital, I-Shou University and all of the experiments were performed in accordance with the guidelines of the Animal Committee and with ethics approval from the Institutional Review Board of E-Da Hospital, I-Shou University, Taiwan (Protocol Number: 0805714). All of the procedures performed on these mice were conducted according to National Institutes of Health guidelines.

### Mouse Partial Hepatectomy Model

The PHx procedure was performed under sterile conditions by the method described previously^1^. This procedure resulted in removal of 70% of the liver. Isoflurane inhalation (Matrx VIP3000, Midmark, Versailles. OH, USA) was used to anesthetize the animals. In the mouse 70% PHx model, the median and left lobes were resected from mice of between 6 and 8 weeks of age. The remaining liver was obtained from defined time points after PHx: 0 h, 6 h, 12 h, 18 h, 24 h, 48 h, 72 h, 120 h, and 168 h. Sham-operated control mice underwent the same surgical procedure without ligation of lobes and without removal of 70% of the liver. Moreover, another PHx model involved the removal of 90% of the total liver.

### Pharmacological modulation of autophagy

In the treatment groups, either amiodarone (30 mg/kg; Sigma, A8423, St. Louis, MO, USA) or chloroquine (CQ; 60 mg/kg; Sigma, C6628, St. Louis, MO, USA) were administered intraperitoneally 30 min before either the control operation or the PHx surgical procedure and were then administered intraperitoneally once per day for 7 days. In the vehicle-treated group, the mice received the same volume of saline intraperitoneally. The dose of amiodarone and CQ used in the mice was chosen according to previous studies^13^,^22^,^30^.

### Small interfering RNA (siRNA) for Atg7 in PHx mice

To inhibit Atg7 expression in murine livers, siRNA was injected (0.7 nmol/g) through the tail vein using the hydrodynamic technique. Forty-eight hours later, the mice were subjected to PHx. Mouse Atg7-specific siRNA (5′ CUGUGAACUUCUCUGACGU[dT][dT] 3′) (Sigma, Oligo #: 8015025863-000020) and negative siRNA (5′ GAUCAUACGUGCGAUCAGA[dT][dT] 3′) (Sigma, Oligo #: 8013122026-000020) were obtained from Sigma-Aldrich (St. Louis, MO, USA).

### Immunohistochemistry

Liver paraffin sections from each sample were cut into 5-μm sections and stained with proliferation marker Ki67 rabbit antibody (Thermo Fisher Scientific, PA5-19462, Waltham, MA, USA) using the avidin-biotin-peroxidase complex technique (Vectastain ABC kit and DAB peroxidase substrate kit, Vector Laboratories, Burlingame, CA, USA). The tissue was counterstained with H&E. The staining was visualized using an Axiovert 40 CFL inverted microscope (Carl Zeiss MicroImaging, Thornwood, NY, USA) and then analyzed. The average hepatocyte proliferation rate was represented by the percentage of Ki67-positive hepatocytes in the total number of hepatocytes in 15 random selected fields taken at 200 × magnification for each group of mice.

### Transmission electron microscopy

The specimens were excised and fixed with fixative buffer containing 2% paraformaldehyde and 2.5% glutaraldehyde in 0.1 M PBS and were stored at 4°C until embedding. Tissue samples were then post-fixed in 1% phosphate-buffered osmium tetroxide and embedded in Spurr’s resin. The samples were cut into 0.12-μm thin sections and stained with 0.2% lead citrate and 1% uranyl acetate. The images were examined using a JEOL TEM-2000 EX II (JEOL, Tokyo, Japan).

### Western blot analysis

The tissues were homogenized in lysis buffer. Lysates were centrifuged at 17,000 × g for 10 min. An aliquot of the supernatant was used to determine protein concentration (Bio-Rad Laboratories, Hercules, CA, USA). Protein aliquots were mixed with 4× lithium dodecyl sulfate sample buffer, electrophoresed on 4–12% SDS-polyacrylamide gels, and transferred electrophoretically onto nitrocellulose paper. The membranes were immunoblotted with the primary antibody. This step was followed by the addition of horseradish peroxidase-conjugated secondary antibody. After the final wash, the membranes were probed using enhanced chemiluminescence (Amersham Pharmacia Biotech, Piscataway, NJ, USA) and autoradiographed. The membranes were immunoblotted with anti-LC3 (Novus Biological, NB100–2220, Littleton, CO, USA), anti-Proliferating Cell Nuclear Antigen (PCNA) (Abcam, ab29, Cambridge, UK), anti-cyclin D1 (GeneTex, GTX61845, San Antonio, TX, USA), anti-cyclin A, B, E, Keap1, and Nrf2 (Santa Cruz Biotechnology, Sc-751, Sc-25764, Sc-481, Sc-33568, Sc-722, Santa Cruz, CA, USA), anti-TGF-β1 (Abcam, ab64715, Cambridge, UK), anti-Caspase-8 (R&D Systems, AF1650, Minneapolis, MN, USA), anti-Caspase-3 (Abcam, ab47131, Cambridge, UK), anti-phosphorylated and total mTOR, anti-phosphorylated and total 4EBP1, anti-phosphorylated and total p70 S6 Kinase (Cell Signaling Technology, #4060, #9272, #2971, #2983, #9205, #2708, Beverly, MA, USA), and anti-β-actin (a loading control) (Novus Biological, NB600-501, Littleton, CO, USA) antibodies. The signal intensity of each protein band was quantitated using National Institutes of Health ImageJ software and was statistically analyzed.

### Reverse Transcription-Quantitative Polymerase Chain Reaction (RT-qPCR)

Total RNA was extracted from liver tissues using the QuickGene RNA tissue kit SII (Fuji Photo Film, Cat. No. RT-S2, Tokyo, Japan), and the procedure was performed according to manufacturer’s instruction. RNA was reverse transcribed using the RevertAid H Minus First Strand cDNA synthesis kit. The qPCR analysis of p62, IL-6, IL-8, and actin mRNA expressions was performed on an Eco Real-time PCR system (Illumina, San Diego, CA, USA) using VeriQuest Fast SYBR Green qPCR Master Mix (Life Technologies, Grand Island, NY, USA). The primers are listed in Supplementary Table S1. The amplification protocol consisted of 35 cycles of denaturation for 15 s at 95°C and annealing and extension for 60 s at 60 °C. All of the samples were amplified with β-actin as an endogenous loading control. The relative expression of each gene was calculated and expressed.

### Staining for Senescence-Associated β-Galactosidase (SA-β-gal) activity

Assessment of SA-β-gal activity in the liver tissues was performed using Senescence Detection kit (Biovision, Mountain View, CA, USA) according to the manufacturer’s protocol.

### Autophagic flux assay

The autophagic flux was determined by immunoblotting with an anti-LC3 antibody (Novus Biological, NB100-2220, Littleton, CO, USA) and conducting a densitometric analysis of LC3-II levels relative to actin (a loading control). The cumulative LC3-II levels were measured in the presence and absence of CQ treatment, which both increase lysosomal pH and impair autolysosome functions. The value of the autophagic flux was quantified as in our previous studies by subtracting the LC3-II levels in mice without CQ treatment from the LC3-II levels in mice treated with CQ^13^,^14^,^28^.

### Quantification of intracellular ATP concentrations

The hepatic ATP concentration was measured using an ATP Detection Reagent kit (Toyo Ink., Tokyo, Japan) according to the manufacturer’s instructions.

### Measurement of mitochondrial membrane potential

Mitochondrial membrane potential (MMP) was evaluated using cationic dye JC-1. In normal cells, JC-1 aggregates in mitochondria, fluorescing red. In apoptotic cells, JC-1 accrues in the cytosol as a green fluorescing monomer. Cells were harvested and then incubated with JC-1 10 mg/mL for 15 minutes at 37 μC in the dark. The cells were harvested, suspended in PBS, and analyzed by flow cytometry.

### Biochemical analysis

Mouse plasma samples were collected, and the concentrations of ALT were determined using GPT-JS kits (Denka Seiken, Tokyo, Japan) and an automated biochemical analyzer (model TBA-200FR; Toshiba, Tokyo, Japan).

### Cytokine analysis

Plasma IL-6 and IL-8 levels were measured using commercially available cytometric bead array (CBA) inflammation kits (BD Biosciences, 558301, San Jose, CA, USA) according to the manufacturer’s instructions.

### Statistical analyses

Quantitative data (mean ± SEM) were subjected to Student’s t test analysis, using SigmaStat 3.5 (Systat Software, San Jose, CA, USA). *P* < 0.05 was considered significant.

## Additional Information

**How to cite this article**: Lin, C.-W. *et al.* Amiodarone as an autophagy promoter reduces liver injury and enhances liver regeneration and survival in mice after partial hepatectomy. *Sci. Rep.* **5**, 15807; doi: 10.1038/srep15807 (2015).

## Supplementary Material

Supplementary Information
