# Supplementary material for: Amiodarone as an autophagy promoter reduces liver injury and enhances liver regeneration and survival in mice after partial hepatectomy
Source: Sci Rep. 2015 Oct 30;5:15807. doi: 10.1038/srep15807 (PMC4626804; doi:10.1038/srep15807)
Supplement: Supplementary Information [file srep15807-s1.doc]

**Amiodarone as an autophagy promoter reduces liver injury and enhances liver regeneration and survival in mice after partial hepatectomy**

Chih-Wen Lin, MD^1,2,3,4^, Yaw-Sen Chen, MD^4,5^, Chih-Che Lin, MD, PhD^8^, Yun-Ju Chen, PhD^6,7^, Gin-Ho Lo, MD^2,4^, Po-Huang Lee, MD, PhD^4,5^, Po-Lin Kuo, PhD^1^, Chia-Yen Dia, MD, PhD^1,9,10^, Jee-Fu Huang, MD, PhD^1,9^, Wang-Long Chung, MD, PhD^1,9^, *Ming-Lung Yu, MD, PhD^1,9,10,11^

**Supplementary Table S1. Reverse Transcription-Quantitative Polymerase Chain Reaction (RT-qPCR) Primer Sequences.**

| Genes | Sequences |
| --- | --- |
| β-actin Sense | CAACTGGGACGACATGGAGAAAAT |
| Antisense | CCAGAGGCGTACAGGGATAGCAC |
| IL-6 Sense | ACCACCGGGCTTCCTAAGG |
| Antisense | CTGTAGGAATGGTGGCCAAAG |
| IL-8 Sense | GAAAGCGCAAGTCCTCAAAG |
| Antisense | TGGGTAGGAGATGGAGATGC |
| P62 Sense | CCAAATGAAGATGAGCATAGGACAT |
| Antisense | GTTGACCTGCAGTCGTTTTGC |
